# Effect of chromatin structure on quantitative ultrasound parameters

**DOI:** 10.18632/oncotarget.14816

**Published:** 2017-01-25

**Authors:** Maurice Pasternak, Lilian Doss, Golnaz Farhat, Azza Al-Mahrouki, Christina Hyunjung Kim, Michael Kolios, William Tyler Tran, Gregory J. Czarnota

**Affiliations:** ^1^ Department of Radiation Oncology, Sunnybrook Health Sciences Centre, Toronto, Canada; ^2^ Physical Sciences, Sunnybrook Research Institute, Toronto, Canada; ^3^ Department of Medical Biophysics, University of Toronto, Toronto, Canada; ^4^ Department of Radiation Oncology, University of Toronto, Toronto, Canada; ^5^ Department of Physics, Ryerson University, Toronto, Canada

**Keywords:** ultrasound, chromatin, spectral analysis, form-factor analysis, electron microscopy

## Abstract

High-frequency ultrasound (~20 MHz) techniques were investigated using *in vitro* and *ex vivo* models to determine whether alterations in chromatin structure are responsible for ultrasound backscatter changes in biological samples. Acute myeloid leukemia (AML) cells and their isolated nuclei were exposed to various chromatin altering treatments. These included 10 different ionic environments, DNA cleaving and unfolding agents, as well as DNA condensing agents. Raw radiofrequency (RF) data was used to generate quantitative ultrasound parameters from spectral and form factor analyses. Chromatin structure was evaluated using electron microscopy. Results indicated that trends in quantitative ultrasound parameters mirrored trends in biophysical chromatin structure parameters. In general, higher ordered states of chromatin compaction resulted in increases to ultrasound paramaters of midband fit, spectral intercept, and estimated scatterer concentration, while samples with decondensed forms of chromatin followed an opposite trend. Experiments with isolated nuclei demonstrated that chromatin changes alone were sufficient to account for these observations. Experiments with *ex vivo* samples indicated similar effects of chromatin structure changes. The results obtained in this research provide a mechanistic explanation for ultrasound investigations studying scattering from cells and tissues undergoing biological processes affecting chromatin.

## INTRODUCTION

The determination of tumor response to treatment is based on the Response Evaluation Criteria in Solid Tumors (RECIST) parameters. Current RECIST criteria solely utilize tumor dimensional size in an assessment every six to eight weeks following a cycle of chemotherapy [1]. The RECIST method has several limitations, including its inability to accommodate responding tumors dying by oncosis, dependence on gross anatomical changes that happen much later than molecular ones, and a failure to account for fibrosis. Several functional imaging techniques have been developed such as PET/CT and MRI to assess tumor response. However, these modalities are often limited by their cost, repeated use of radioactive material (for PET/CT), and their primary use to measure standard anatomical features. In contrast, ultrasound is an imaging technique that does not utilize ionizing radiation, is low cost, label-free, features real-time imaging, and provides relatively high-resolution images. These qualities make it a superb candidate technique for multiple imaging sessions per patient. For these reasons, quantitative ultrasound in the 2-10 MHz range already has multiple applications in pathological breast assessment, and initial cancer detection [2, 3, 4]. Furthermore, advances in the engineering of ultrasound probes have permitted for the use of 20 MHz frequencies and higher in the clinic. Within this work, the term “high-frequency” will refer to the use of ultrasound probes with central frequencies of 20 MHz or greater, as used in previous studies [5, 6].

Ultrasound imaging operates by detecting the effects of changes to the physical characteristics of tissues, such as their acoustic impedances (Z= √(ρ/κ), where ρ is the density and κ is the compressibility), sizes, and the spatial distribution of scatterers change as a function of treatment. Furthermore, it has been suggested that the information contained in ultrasound radiofrequency (RF) signals is related to these acoustic and structural properties of tissue [7, 8]. The RF data can be analyzed to extract meaningful parameters, most commonly the mid-band fit, spectral slope, and spectral intercept, which are related to acoustic scatterer distribution, size, and the concentration of acoustic scatterers [9, 10]. These parameters are derived from linear regression analysis of a normalized backscatter power spectrum. Such quantitative ultrasound analyses have been used to characterize different tissue types including eye, liver and prostate tissue and various different tumors [9, 10, 11, 12]. In addition, this methodology has been adapted to the detection of treatment responses [2].

Prominent biophysical changes are known to characterize nuclear changes during cell death, which has driven the hypothesis that nuclear chromatin plays a definitive role in acoustic scattering for studies investigating cell death through ultrasound techniques [13]. The first series of experiments to suggest this was an investigation by Sherar et al. [14], where increased backscatter using 100 MHz ultrasound was shown to correlate to hypoxic regions inside tumor spheroids containing pyknotic nuclei. Experiments with apoptotic cells with condensed and fragmented nuclear material demonstrated for the first time the detection of cell death using high-frequency ultrasound and a dependence on changes in nuclear material [13, 15]. Further work demonstrated that the increased ultrasound backscatter in mitotic populations containing condensed chromatin could be reversed by the addition of DNase I [13]. A more recent study determined that cell death can be quantitatively correlated to spectral ultrasound parameters on the basis of chromatin bodies formed inside paclitaxel-treated cells during cell death [16]. Other work has demonstrated that the backscatter increased from isolated apoptotic nuclei compared to nuclei from viable cells accounted for the magnitude of backscatter increase from apoptotic cells compared to viable cells [17].

Apart from cell death mechanisms, other factors are known to influence chromatin structure and folding. Under hypertonic conditions, nuclear material is known to condense [18]. This mechanism is a result of chromatin sensitivity to ions such as sodium involved in molecular interactions required to maintain physiological structure. Perturbing the balance of ions towards either hypotonic or hypertonic conditions alters electrostatic forces involving the phosphate backbone and associated proteins [19]. Alternatively, post-translational modifications can exhibit similar effects on chromatin structure. In particular, sodium butyrate is a chemical known to alter chromatin structure by non-competitively inhibiting histone deacteylases, resulting in highly acetylated chromatin that takes on a less-compact conformation [20, 21] associated with increased gene expression.

In this study, quantitative ultrasound techniques were utilized to study the effect of structural states of chromatin on scattering parameters using several treatment conditions that induced different degrees of chromatin compaction. Acute myeloid leukemia (AML5) cells were used in this study because of the well characterized response of this cell line to a cisplatinum induction of chromatin condensation in regards to ultrasound parameters. It has also been determined that *in vitro* preparation methods for ultrasound studies of this cell line do not influence final ultrasound results [15]. Cells or isolated nuclei were subjected to over 500-fold range differences in sodium chloride concentrations and to other chromatin-altering treatments including sodium butyrate, DNase I digestion, exposure to colchicine, and cisplatinum. DNase I treatment was also repeated for a more complex *ex vivo* mouse liver model. Results indicated that mid-band fit trends were linked to changes in chromatin compaction. All conditions inducing less dense states of chromatin resulted in decreased midband fit, spectral intercept, and estimated acoustic concentration. Vice versa, all conditions producing more condensed states of chromatin caused increases in these ultrasound parameters markers.

This study provides evidence indicating that chromatin is a major scatterer of ultrasound and that the degree of its compaction has a significant influence on ultrasound parameters. This provides a mechanistic explanation for ongoing observations in ultrasound studies in which cellular states involving changes in chromatin structure are linked to changes in ultrasound backscatter.

## RESULTS

**Figure 1:**
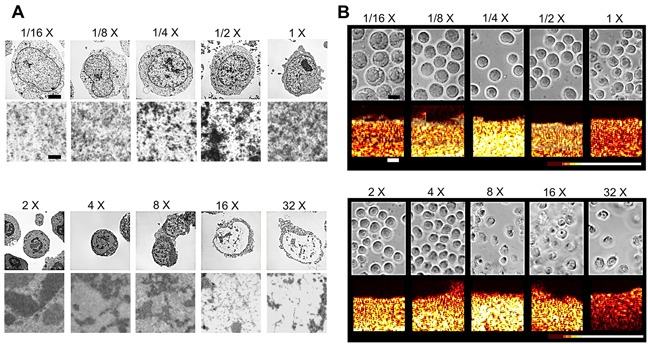
**A**. Representative electron microscopy images of fixed AML-5 cells subjected to varying concentrations of sodium chloride. Top row panels depict whole cell morphology. Bottom panels depict chromatin strucutre at high magnification for each salinity. The scale bar in the top row represents 2 microns. The lower scale bar represents 100 nm. **B**. Light microscopy (top row) and corresponding color-coated B-mode ultrasound images of non-fixed cell samples. Cell and nuclear size changes differ from electron microscopy due to no fixation step prior to imaging. Speckle intensity is illustrated through pixel color, with dark red representing less scattering and white representing increased scattering. The scale bar in light microscopy images represents 6 microns. The scale bar in ultrasound B-mode images represents 1 mm.

Transmission electron microscopy (TEM) indicated that sodium chloride concentration had a significant effect on the structure of chromatin (Figure 1A). Initial increases in salinity to 2X, 4X, and 8X physiological salinity resulted in the formation of visible high-order chromatin clusters. However, by 16X physiological salinity, these clusters decreased significantly in number and size as less-compact 10 nm chromatin fibres become discernable. In general, similar trends were observed when decreasing sodium concentration, where $\frac{1}{2}$X and $\frac{1}{4}$X salinities were marked by visible aggregation of chromatin, indicating an increase in compaction. Further dilution to $\frac{1}{8}$X and $\frac{1}{16}$X salinities displayed significant decondensation relative to the control (Figure 1A). Light microscopy images corresponded very well to TEM data (Figure 1B) indicating changes in nuclear morphology and size. Some discrepancies in cell size trends between the two imaging modalities stem from the effect of fixation on cell and nuclear volume. As ultrasound data was acquired from non-fixed samples, size trends from light microscopy images are more accurate of physiological response. In ultrasound data, B-mode speckle intensity increased for salinities featuring compact forms of chromatin versus lessened intensity for salinities featuring decondensed structures. Light microscopy revealed expected outcomes for cellular of nuclear sizes as a function of salt concentration. Namely, cells and nuclei at hypertonic conditions tended to decrease in size while hypotonic conditions induced swelling and increases in size (Figure 1B).

**Figure 2:**
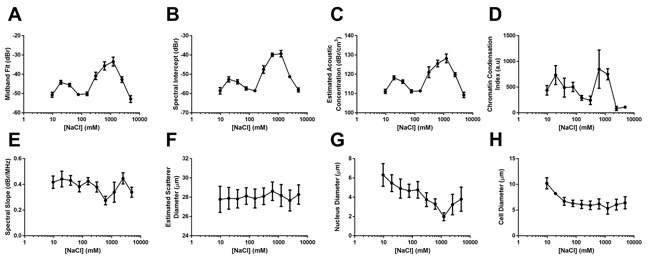
Quantitative data dervied from spectral ultrasound analysis, electron miscropy analysis, and ultrasound form factor analysis Results of relative **A**. mid-band fit, **B**. spectral intercept, and **C**. estimated acoustic concentration for whole cells subjected to sodium concentrations from 1/16X salinity to 32X salinity. Observed trends corresponded strongly to an **D**. index of chromatin condensation based on quantifying the number of 30 nm strands and larger chromatin clusters per high-powered field and multiplying by a scaling factor for clearer data presentation. **E**. Results from the spectral slope parameter were not signficantly different, corresponding to statistically-similar values for **F**. estimated scatterer diameter throughout all salinities. **G**. Measured sizes of nuclear diameter and **H**. cellular diameter did demonstrate trends of increasing size for lower salt concentrations, but did not appear to affect spectral slope or estimated scatterer diameter. Error bars represent SD at n ≥ 4.

Quantitative ultrasound data demonstrated that changes to acoustic parameters such as midband fit (MBF, Figure 2A) corresponded in general to changes in the degree of chromatin compaction. Specifically, at elevated salinities the MBF increased by 9.3 ± 2.1 dBr, 14.5 ± 2.3 dBr, and 16.7 ± 2.5 dBr for 2X, 4X, and 8X physiological sodium concentration, respectively. Relative to the 8X salt concentration, 16X and 32X salinities were marked by decreases of 9.3 ± 2.7 dBr and 19.4 ± 2.9 dBr, respectively. The latter 32X salinity featured a MBF value 2.7 ± 2.0 dBr lower than the control. At these ionic environments the nuclear material appeared disaggregated with individual 30 nm DNA fibres more obvious.

In the hypotonic direction, the MBF increased by 4.6 ± 1.3 dBr and 5.9 ± 1.4 dBr for $\frac{1}{4}$X and $\frac{1}{8}$X salinities before dropping back down to statistically-similar values relative to the control at $\frac{1}{16}$X salinity. Similar trends were obtained for the spectral intercept and estimated acoustic concentration parameters (Figure 2B and 2C). Trends in chromatin condensation (defined by the number of 30 nm fibre clusters and >50 nm compact aggregates as assessed from nuclear structure using TEM) paralleled those of these quantitative ultrasound parameters (Figure 2D). For these experiments, the spectral slope and estimated scatterer diameter (Figure 2E and 2F) revealed relatively large variances resulting in changes overall that were not statistically significant despite measured differences in nuclear and cell diameters (Figure 2G and 2H).

In order to test for possible effects of the cytoplasm and its contents being responsible for the observed changes in ultrasound spectral and form factor parameters, salinity experiments were repeated for isolated nuclei (Supplementary Figure 1). Most trends remained consistent with the results from whole cell ensembles. This held true for midband fit, spectral intercept and the acoustic scatterer concentration. However, it was notable that in the hypertonic direction, the sudden decrease in MBF and spectral intercept parameters occurred at 8X salinity for isolated nuclei as opposed to 16X for whole cells. This decrease was significantly greater for isolated nuclei, as final MBF values for 8X salinity nuclei were 9.3 ± 0.5 dBr lower than the 1X control. In addition, at this 8X salinity, the spectral slope significantly increased for isolated nuclei samples, indicating a considerable decrease in scatterer size (Supplementary Figure 1). Further form factor analysis of the estimated scatterer diameter did reveal changes that were statistically significantly different from control samples at the 8X sodium chloride concentration (Supplementary Figure 1). Moreover, changes were in parallel to that observed for nuclear size from light microscopy.

**Figure 3:**
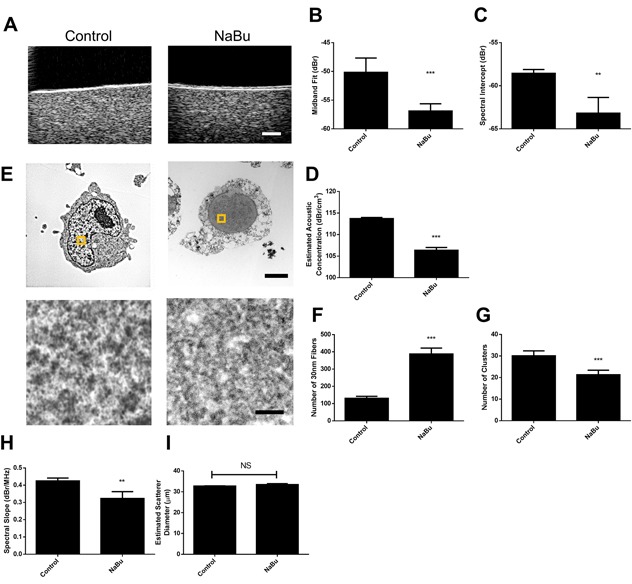
Representative results from sodium butyrate treatment of *in vitro* samples **A**. B-mode images indicated decreases in ultrasound backscatter, corresponding to decreases in **B**. midband fit, **C**. spectral intercept, and **D**. estimated acoustic concentration. **E**. Electron microscopy images depict signficant alterations in chromatin structure, indicating a decrease in chromatin compaction. Selected regions of higher maginifcation are represented by squares on lower magnificaion panels. **F**. Quantified counts of the number of 30 nm strands and **G**. larger chromatin clusters per high-powered field. **H**. Spectral slope and **I**. estimated scatterer diameter measurements demonstrated slight changes as a function of treatment. ** and *** indicate p<0.01 and p<0.001, respectively, for n ≥ 4 samples. The scale bar in B-mode images represents 1 mm. Scale bars for low-magnification and high-magnification electron micrscopy images correspond to 2 βm and 100 nm, respectively.

Exposure of cell samples to sodium butyrate produced repeatable changes to ultrasound scattering and the derived mid-band fit spectral parameters (Figure 3). Representative B-mode images from a sodium butyrate treated cells demonstrated an obvious decrease in speckle intensity (Figure 3A). This translated into quantitative ultrasound parameters. Specifically, midband fit, spectral intercept, and acoustic concentration decreased by 6.7 ± 1.2 dBr, 4.6 ± 1.9 dBr, 7.36 ± 0.69 dBr/cm^3^, respectively. These parameters were statistically significantly different (p<0.01) in comparison to untreated control cells (Figure 3B–3D).

Analysis of nuclear ultrastructure using TEM linked these observations to the structure of chromatin. As expected from hyper-acetylated chromatin, the degree of chromatin condensation was visibly decreased (Figure 3E). When quantified, the number of larger and darker-staining >50 nm chromatin clusters significantly decreased per high-magnification field (Figure 3F) while the number of the relatively less-condensed 30 nm fiber structures increased (Figure 3G), suggesting that the former structures decondensed into the latter. Spectral slope did demonstrate a small decrease of 0.10 ± 0.04 dBr, but this did not translate to any changes in average acoustic diameter extracted from the Fluid-filled sphere model (Figure 3H and 3I).

**Figure 4:**
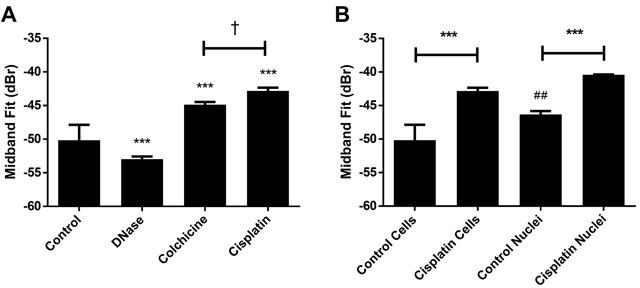
Representative mid-band fit data from other chromatin-altering treatments **A**. Results from DNase I, colchicine and cisplatinum treatments, indicating that conditions inducing chromatin condensation were sufficient to increase midband fit. A significant difference was determined to exist between colchicine and cisplatinum treatment. **B**. Results from isolated nuclei experiments involving cisplatinum treatment. Both isolated nuclei and whole cells demonstrated increased midband fit values after exposure to cisplatinum. Untreated control nuclei demonstrated a significantly higher midband fit value than untreated whole cells. *** indicates p<0.001 between the indicated condition and the corresponding untreated control. † indicates p<0.05 significance between colchicine and cisplatinum treatments. ## indicates p<0.01 significance between untreated nuclei and untreated whole cells. n ≥ 4 for all conditions.

In order to test other chromatin alteration, treatments using DNase I exposure to cut chromatin fibres in order to cause unravelling were used. Cells with condensed nuclear material were prepared using colchicine to arrest cells at metaphase of mitosis. Cell samples with condensed and aggregated nuclear material were also prepared after exposing growing cells to cisplatinum to induce apoptosis. DNase I lysis of chromatin resulted in a decrease of 2.8 ± 2.3 dBr relative to the untreated control. Treatment with colchicine increased the population of mitotic cells containing condensed and non-fragmented chromatin and led to a 5.3 ± 2.3 dBr increase in MBF. In contrast, cisplatinum-induced condensation and fragmentation of chromatin increased MBF by 7.3 ± 2.3 dBr (Figure 4A). Notably, the difference in absolute MBF value increase between these latter two treatments is a significant 2.0 ± 0.6 dBr, suggesting additional effects of coupling fragmentation of nuclear material beyond simple nuclear condensation.

Nuclei were also isolated from cisplatinum treated cells that had been rendered apoptotic [15] (Figure 4B). Trends remained consistent in comparison to work from cells, where MBF increased by 5.9 ± 0.5 dBr for nuclei from cisplatinum-treated cells versus untreated control nuclei. Data also revealed that untreated isolated nuclei on their own displayed a statistically-significant 4.0 ± 2.6 dBr greater MBF than untreated whole cells. Additional time-of-flight measurements were performed to ascertain the speed of sound in samples subjected to different salt concentrations or chromatin-altering treatment (Supplementary Figure 2). No significant changes were found to occur in this parameter for any treatments above.

**Figure 5:**
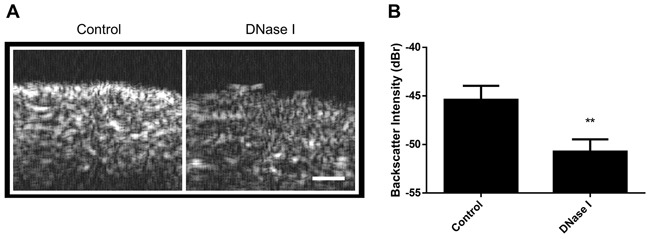
Imaging of DNase I treatment in excised mouse liver **A**. B-mode images of control (left) and 1 hour DNase I- treated samples (right). Darker pixels correspond to lower levels of ultrasound scattering and white representing regions of increased ultrasound scattering levels. **B**. When quantified to determine backscatter intensity, DNase I treatment results in signficant decreases to sample echogenecity. ** indicates p<0.01 statistical significance for n=4 measurements. Scale bar represents 0.5 mm.

In order to test for whether similar acoustic observations hold for more complex models, DNase I treatment was also used with excised mouse livers. Following treatment, these livers demonstrated decreases in acoustic echogenicity similar to observations in cell samples, indicating effects of nuclear structure despite increased model complexity (Figure 5A). The backscatter intensity decreased by 5.3 ± 1.9 dBr with 30 minutes of DNase I digestion (Figure 5B).

## DISCUSSION

The results presented in this study provide significant support to the working hypothesis that nuclear chromatin in cells is a scatterer of high-frequency ultrasound (≥20 MHz), providing a mechanistic explanation for the observations in multiple earlier ultrasound studies assessing chromatin-altering biological processes such as apoptosis. Modulation of chromatin effects on acoustic properties in the study here, was detected by ultrasound-based spectral biomarkers (MBF and spectral intercept parameters) and by form factor biomarkers (estimated acoustic concentration and acoustic scatterer diameter). The results were confirmatory across *in vitro* and *ex vivo* studies where AML-5 cell line and excised mouse liver samples underwent various chromatin-altering treatments. Several previous studies have supported the possibility of nuclear material playing an important role in changes to ultrasound backscatter and other biophysical parameters [13, 16, 22]. The study here is the first direct investigation of the effects of nuclear structure on ultrasound backscatter and supports the use of quantitative ultrasound analysis in evaluating biological processes involving chromatin configuration changes.

Ionic environment-based experiments revealed changes in spectral and form factor parameters that were linked to nuclear structure. Biological correlates were obtained using transmission electron microscopy. In particular, samples demonstrating higher-order compaction of chromatin were marked by grossly increased backscatter signal intensity in corresponding B-mode images and changes in ultrasound spectral parameters. Conversely, samples that demonstrated a reduction of chromatin compaction featured lower speckle intensity values. This occurred both at higher and lower ionic environments. This was particularly evident in the transition between 8X and 16X sodium concentration, as chromatin was found to decondense into 10 nm fibres at the latter salinity. Confirmatory trends were also observed in the direction of decreasing sodium concentration, where the transition to $\frac{1}{16}$X salinity was marked by decreases in signal intensity alongside observable chromatin de-compaction. This indicated a reasonable sensitivity of the ultrasound technique to assessing chromatin compaction status.

Changes in the spectral biomarkers midband fit and spectral intercept also corresponded very well to the observations here. Values increased in parallel to the speckle intensity of ultrasound images. This was concordant with trends in quantified chromatin condensation apparent in high-magnification TEM images. The chromatin condensation index, based on quantifying the number of higher-order chromatin structures present in representative cell samples from each environment, followed a similar trend. Other studies investigating the structure of chromatin at different NaCl concentrations were in agreement with several observations presented here. The higher condensation values around $\frac{1}{8}$X salinity (19.25 mM NaCl) have been related to lessened electrostatic repulsion between linker DNA segments and transition from the loose zig-zag morphology to condensed fibre-like structure in isolated polynucleosomes [23]. The sudden decrease in spectral parameters and chromatin condensation at $\frac{1}{16}$X salinity (9.63 mM NaCl) can be attributed to the greater abundance of uncondensed chromatin observed around lower sodium ion concentrations as indicated by linear dichroism studies [23, 24]. For higher salinities, there is a well-known phenomenon of supercoiling that occurs during which 30 nm fibers further aggregate into higher-order structures [24]. Considering the composition of chromatin being approximately 50% DNA (density of 1.71g/cm^3^) and 50% protein (density of 1.35g/cm^3^) [25] and that the speed of sound going through DNA of varying conformation ranges from 1900 to 2400 m/s [26, 27], these structures likely demonstrate a significantly higher impedance relative to the surrounding media, resulting in greater ultrasound scattering. Isolated nuclei data in this study strongly support this hypothesis, as a 4.0 ± 2.6 dBr increase to MBF was observed for nuclei samples versus whole cells, indicating that chromatin on its own scatters ultrasound more at the frequency used in this study.

Increases of salinity into the range of 16X and 32X physiological NaCl concentration demonstrated the formation of decondensed chromatin such that 10 nm fibres were evident. In parallel, spectral parameter values decreased significantly to reflect lessened ultrasound scattering. These observations were in agreement with previous studies that marked histone (H3-H4)_2_ tetramer dissociation occurs at high NaCl concentrations, which indicates a denatured and unwound state of chromatin [28]. These observations were recapitulated for isolated nuclei samples, with the additional observation that the decrease in chromatin compaction and corresponding MBF and spectral intercept parameters occur at an earlier 8X NaCl concentration (Supplementary Data). The decrease in these parameters was also significantly greater in isolated nuclei than for whole cells. Considering the absence of the cytoplasm and plasma membrane that normally serve as active buffers and barriers to salt perturbations [29], it would be expected for nuclei to respond earlier to elevated salt levels and to a greater degree. Moreover, the 8X (1232 mM) sodium concentration at which this occurred is well above the 900 mM salinity at which H2A-H2B histone dissociation was previously observed in isolated calf thymus nuclei [28].

With regards to changes in cell size observed in light microscopy images, apparent changes in nuclear and cell size were difficult to translate into statistically significant trends due to a competing effect between nuclear and cytosolic environments. Namely, changes to the ionic environment would impact nuclear structure and nuclear size, but would be balanced by the effects of the cell cytosol, which buffered the impact of changing sodium concentration. Upon repeating experiments in isolated nuclei, a lateral shift in spectral slope trend was observed (Supplementary Data).

Experiments involving treatment with sodium butyrate matched predictions based on the hypothesis that chromatin compaction influences ultrasound scattering. Results indicated that higher-order condensed bodies unfolded to produce more of the relatively-less condensed 30 nm fibres and that this change was reflected through decreases in MBF and spectral intercept parameters. This suggested that sample-wide bulk decreases in chromatin condensation are detectable by ultrasound. Furthermore, results from mouse liver treatments where DNA was digested demonstrated similar trends to those of the *in vitro* model, indicating that static extracellular structures such as the surrounding protein matrix contribute minimally to changes in ultrasound during DNase I digestion of nuclear material. This agrees with observations from other studies noting that backscatter properties can be similar between highly-cellular xenograft tumors versus *in vitro* cell models that mimic tumor cell packing [30], despite the increased complexity of the former model.

Several previous investigations of ultrasound analysis of biological tissue support the results presented here. In particular, the induction of chromatin condensation in apoptosis has led to increases in ultrasound backscatter signal intensity at various frequency ranges in previous studies [13, 53]. This has not been limited to apoptotic cell death, as post-mitotic death has also been characterized and a quantitative relationship has been found to exist between the midband fit spectral parameter and the cell death index when defined by the presence of condensed chromatin bodies inside dying cells [16]. In the study here, chemotherapeutic treatments of colchicine or cisplatinum induced chromatin condensation versus condensation-fragmentation in cell samples, respectively. A previous investigation of such treatments only measured increased backscatter [13]. This study confirmed that quantitative parameters, including midband fit and spectral intercept, increased as well with these treatments, which was consistent with other results [31]. Another study has also assessed the effect of cisplatinum at multiple time points using conventional-frequency ultrasound and confirmed that the increase in midband fit extends up to 48 hours of treatment inducing chromatin changes [32]. Additionally, while these results suggest that chromatin condensation was sufficient to increase these spectral parameters, having them fragment into additional structures resulted in a further 2.0 dBr greater midband fit. Ultrasound theory predicts that midband fit would be affected not only by the relative impedance of scatterers, but also their concentration and location to one another [8, 33]. It is plausible that the additional nuclear fragmentation occurring in response to cisplatinum treatment, but not colchicine, would increase scatterer concentration and randomization, translating to an even more elevated midband fit as observed.

It may be argued that spectral changes may be originating from the cytoplasm and that other organelles such as mitochondria may be significant scatterers of ultrasound besides chromatin. However, when cisplatinum treatment was repeated and nuclei isolated, increases in midband fit were similar to those observed in experiments involving whole cells. While this does not dismiss the potential of other organelles to serve as scatterers of ultrasound, the observations in this study suggest that the main source of scattering is present in the nucleus, hypothesized to be chromatin. There are additional components of nuclei that may play a role. In particular, it is known that the nucleoskeleton influences nuclear mechanical properties such as compressibility and elasticity [34], which in turn have an effect on acoustic impedance. Therefore, it would be prudent for additional ultrasound investigations to determine whether nucleoskeletal components such as lamin-A [35] change in the course of salt concentration alteration or any of the other chemical treatments implemented here.

The study here also used higher order models to investigate ultrasound backscatter permitting estimation of acoustics scatterer concentration and diameter. Here, form factor analysis using the Anderson Fluid Filled Sphere model, which has been previously described [56, 36], revealed that spectral intercept and midband fit changes resulted primarily from increases to acoustic scatterer concentration and not necessarily increases to scatterer diameter. Presumably, the formation of higher forms of chromatin compaction may result in the appearance of more scatterers within the confined volume of the nucleus, interpreted as an increase in scatterer concentration. However, it is important to note that scatterer randomization and relative impedance also play significant roles in determining the degree of spectral scattering [33]. Regarding the former, simulations by Hunt et al. have demonstrated that even under conditions of unchanging scattering strength from nuclear material, the processes of condensation and/or fragmentation increase ultrasound backscatter by allowing for a greater randomization of scatterer positioning [37]. Moreover, given the changes in structure between 10 nm fibres, 30 nm fibers, and larger aggregates, it may be reasonable to assume that significant changes to acoustic impedance follow these alterations. Therefore, while the results suggest that changes to chromatin mechanical and structural properties are related to differences in ultrasound scattering, there is no definitive conclusion on whether this is primarily based on increasing the number of scatterers, their relative impedance, or the degree of randomization. Considering ultrasound theory, it is likely that a combination of these factors are responsible.

Previous use of DNase I on colchicine-treated populations was found to reduce backscatter to that of control populations [13]. It was therefore hypothesized and confirmed in this study that sole DNase I treatment should bring spectral parameters to values below that of untreated controls. Those results were in agreement with the hypothesis that chromatin is a major scatterer of ultrasound, as the enzymatic lysis of chromatin by DNase I would decrease overall chromatin compaction and lead to a lessened ability to scatter interrogating ultrasound. Data from experimentation *ex vivo* revealed a similar trend with exposure of tissue to DNase I. This also held true in experiments *ex vivo* in the work here with excised mouse liver exposed to DNase I.

The observation that chromatin is a structurally dynamic entity is not limited to ultrasound studies, as multiple biochemical investigations have come to similar conclusions [38, 39, 40]. Both intrinsic fluorescence and circular dichroism studies conducted at NaCl concentrations of 550 mM, 950mM, and 1450 mM have observed transitions in nucleosome structure [41]. Other fluorescence studies labelling chromatin nucleosomes at methionine 84 of histone H4 subunits have observed 5 structural states in the range of 0.1 mM to 1000 mM NaCl. Upon further analysis of amino acid separation distance, these states were confirmed to differ significantly in their degree of compaction [42]. Alternative approaches using histone H3 cysteine-110 fluorescent sulfhydryl dyes indicated that between the range of 100 mM and 400 mM NaCl, histones compact significantly before separating at higher monovalent salt concentrations, as observed in this study [43]. While it may be argued that crystallographic studies have resulted in only one static form of isolated chromatin nucleosome, it has been hypothesized that this observation is the likely result of having nucleosome histone octamers crystalize in high monovalent salt conditions corresponding to 4.5 M ammonium sulphate [44]. The majority of these chromatin studies have been carried out while studying the effect of Na^+^, as done in this ultrasound investigation. However, it should be noted that K^+^ ions predominate in the nucleus under physiological conditions and that K^+^ ions have significantly different hydration energies, ionic mobilities, and hydrated radii, all of which may impact chromatin differently relative to Na^+^ [45]. Despite this no significant differences were found between Na^+^ and K^+^ in terms of inducing conformational changes in chromatin nucleosomes, as assessed by fluorescence measurements [40].

This study provides significant additional evidence that chromatin is a major scatterer of ultrasound and that alterations involving chromatin may be detected through the quantitative spectral analysis techniques used here. Regardless of the means of induction, condensation of chromatin into higher-ordered forms resulted in increases to midband fit, spectral intercept, and estimated scatterer concentration parameters. Conversely, methods that reduced chromatin compaction, whether by lysis, hyperacetylation, or salt-based denaturation, resulted in decreases to these parameters. This work provides mechanistic proof for earlier analyses and forms the basis for further application of ultrasound in accessing biological processes that feature changes to chromatin structure – most prominently cancer cell death. As a greater fraction of tumor tissue undergoes cell death, a greater degree of chromatin condensation and possibly fragmentation is expected to occur [13], which this study has demonstrated to result in increases to ultrasound parameters such as midband-fit. Therefore, by extension, such parameters may prove to be effective biophysical markers of cell death response. Indeed, both *in vitro* and *in vivo* ultrasound investigations have successfully correlated ultrasound parameters to cell death on the basis of chromatin status [16, 53]. Furthermore, preliminary use of quantitative ultrasound techniques has permitted for the differentiation between patient responders and non-responders [46, 47]. In a clinical setting, this use of ultrasound would be invaluable to the assessment of treatment efficacy in a non-invasive, real-time, and cost-efficient manner.

## MATERIALS AND METHODS

### Cell culture

Acute myeloid leukemia (OCI-AML-5) cells were derived from a leukemia patient and kindly provided by Dr. Minden (Princess Margaret Cancer Centre, Toronto, ON) and were cultured in 150mL of α-minimal-media (Invitrogen Canada inc., Burlington, Canada) supplemented with 5% fetal bovine serum and 1% Pen-Strep, followed by incubation in 150 mL T-flasks at standard 37°C and 5% CO_2_. For each experimental condition, 1×10^7^ cells were cultured and separated into two experimental groups. 5×10^6^ cells were used to create a cell pellet for acoustic measurement and the other 5×10^6^ cells were used to create a parallel pellet for transmission electron microscopy analysis. Cell pellets of heights 4 mm and diameters of 1 cm were prepared through transferring batched cells to 50mL centrifuge tubes, followed by centrifugation at 2000g for 10 minutes. Consecutively, medium was aspirated and cells were washed with phosphate-buffered saline (PBS). A subsequent round of centrifugation at 2000g and 10 minutes produced the desired cell pellets. This cell line was chosen for its relatively-high growth rate and simple handling to provide adequate quantities of aggregated cells. The use of centrifuged cells serves as an approximation of cell-dense tumours and preparation does not impact on cellularity nor ultrasound characteristics [15].

### Nuclear isolation

Nuclei were isolated to test whether ultrasound backscatter changed in response to different treatment conditions when the effect of the cell cytoplasm was not present. Cell samples (AML) were washed with PBS (Mg^2+^– / Ca^2+^–) followed by centrifugation at 2000g for 10 minutes. Cells were then resuspended in Reticulocte Standard Buffer (RSB) at 20 times the volume of the cell pellet [48]. This hypotonic solution induced swelling and disruption of the cellular membrane. Swollen cells were placed in an ice bath for 10 minutes and subsequently centrifuged at 600g for 5 minutes. Following, a second wash with 0.02% NP40 (a detergent used to wash away cellular membrane remnants in RSB), successful isolation of nuclei was confirmed through bright field light microscopy. A final centrifugation at 500g for 5 minutes was carried out to create the analyzed nuclear samples.

### Cell treatments

To test the hypothesis that structural changes in the nucleus are prominent ultrasound scatterers, various treatments altering chromatin conformation were administered and samples were subsequently imaged using HFUS. Treatments included the use of cisplatinum, colchicine, DNase I, sodium butyrate, or different sodium chloride concentrations.

To induce classical apoptosis, cisplatin, a DNA intercalator that causes p53-dependent apoptosis [49], was administered at a concentration of 10 βg/mL for 24 hours. Cells were examined using light microscopy to confirm that cells underwent cisplatin-induced apoptosis. Isolated nuclei were also exposed to cisplatin at 10 βg/mL for 24 hours and compared to control untreated isolated nuclei, cisplatin-treated cells, and control untreated whole cells.

In a separate treatment, mitotic arrest was induced through incubation with colchicine, a chemical agent commonly used to inhibit microtubule formation [50]. Cells were incubated with colchicine at an end concentration of 0.1 βg/mL for 24 hours. As with other treatments, light microscopy was used to confirm the presence of cells undergoing mitotic arrest.

In order to decrease nuclear density, DNase I was used at a concentration of 15,000 U/mL and incubated at 37°C for 30 minutes with isolated nuclei in order to digest nuclear material. The reaction was arrested through addition of EDTA to a final concentration of 15 mM. The use of 30 minutes of treatment time for this treatment condition as opposed to 24 hours was done to avoid tissue homogenization and restrict the effect of DNase I solely on chromatin lysis.

A decrease in the density of the nucleus was also accomplished through sodium butyrate (NaBu), a toxic compound that promotes the unwinding of chromatin through the inhibition of histone deacetylases [20]. Cell batches were treated up to a final concentration of 2.5 mM of NaBu for 24 hours. Preliminary tests confirmed that for this concentration and treatment duration, chromatin appears to be unwound.

Separately, whole cells and isolated nuclei were immersed in different sodium chloride concentrations (9.6 mM, 19.3 mM, 38.5 mM, 77 mM, 154 mM, 308 mM, 616 mM, 1232 mM, 2464 mM, and 4928 mM, corresponding to $\frac{1}{16}$X, $\frac{1}{8}$X, $\frac{1}{4}$X, $\frac{1}{2}$X, 1X, 2X, 4X, 8X, 16X, and 32X physiological salt concentrations) that led to either increases or decreases in chromatin compaction. Notably, isolated nuclei were restricted to the range between and including $\frac{1}{4}$X and 8X physiological salinity, as further increase or decrease of salt concentration resulted in the complete dissolution of isolated nuclei.

### Ex vivo DNase I liver treatment

This investigation has been conducted in accordance with the ethical standards and according to the Declaration of Helsinki and according to national and international guidelines and has been approved by the authors’ institutional review board.

SCID mice (20-25g) were euthanized by exposure to 100% CO_2_ for 5 minutes. Livers were immediately excised by surgery and immersed in 3 washes of phosphate buffered saline (PBS). Following, livers were either incubated in PBS for 1 hour (control) or in the presence of DNase I for digestion. Specifically, liver tissue was incubated with DNase I at a concentration of 15,000 U/mL at 37°C for 30 minutes. The reaction was arrested through addition of EDTA to a final concentration of 15 mM, leading cells to display significant, but incomplete, dissolution of chromatin.

### Ultrasound imaging

Ultrasound imaging and RF-data acquisition was performed with a high frequency ultrasound device (VS40B, VisualSonics Inc., Toronto, Canada). A single-element transducer with a center frequency of 20 MHz and 16 mm focal depth was used for the experiments (VisualSonics Inc., Toronto, Canada). Bandwidth values for gating RF data within a frequency range were obtained from the power spectrum from a quartz disk reference immersed in PBS. For data analyses (below) a Gaussian-fitted function was used to determine the frequency range covering -6 dB relative to the maximum decibel value of the signal. B-mode images were acquired alongside RF data. RF Data was acquired with a 200 MHz sampling frequency.

For cell studies, custom made polished stainless steel wells were used to centrifuge samples, as done previously [16, 51]. Samples were prepared and used for experiments in triplicate as a minimum. Each experiment included at least one untreated control. For each scan, 140 RF scan lines were acquired from a minimum of four different scan planes separated by at least 250 μm and averaged to reduce noise. The 250 μm distance is larger than the beam width of the ultrasound transducer, ensuring that scan planes in each sample did not overlap. Recorded RF segments were 4 mm, enough to contain the entirety of pellet signal. All acquired data was set with the focus of the transducer adjusted to the center of the pellet, 2 mm below the sample surface. All centrifuged cell samples and livers were scanned in PBS at room temperature.

Frequency domain spectral analysis was performed using normalized power spectra of RF signal with an in-house software in MATLAB (Mathworks, Massachusetts, USA) [52, 53]. Spectral analysis first involved acquiring the normalized amplitude line spectrum, *A_l_*(*f, z_l_*), of an RF line *z_l_* (Eq. 1). This involved taking the ratio of the sample amplitude spectrum, *A_s_*(*f, z_l_*) divided by the reference amplitude spectrum, *A_r_*(*f, z_l_*), after both have undergone a fast Fourier transform and gating with a Hanning window.

$$A_{l}\left(f,z_{l}\right)=\frac{A_{s}(f,z_{l})}{A_{r}(f,z_{l})}$$ [1]

Through this normalization, system-dependent characteristics were removed from the signal [54]. Following this, the log power spectrum, *S*(*f*), was acquired by averaging the squared magnitudes of these normalized amplitude spectra, applying a correction factor for attenuation, $e^{-4(\alpha_{s}-\alpha_{r})(R+\frac{\Delta z}{z})}$, and applying a log function to the result, as summarized in Eq. 2.

$$S\left(f\right)=\log_{10}\frac{1}{N}\overset{N}{\underset{l=1}{\sum }}{\left|A_{l}\left(f,z_{1}\right)\right|}^{2}e^{-4(\alpha_{s}-\alpha_{r})(R+\frac{\Delta z}{z})}$$ [2]

For the attenuation compensation terms, *α_s_* and *α_r_* are the coefficients for the sample and reference data, respectively, R is the axial distance from the transducer to the proximal edge of the ROI window, and *Δz* is the axial window length.

The normalized log power spectrum is a quasi-linear function, making linear regression analysis appropriate for the determination of mid-band fit (MBF), spectral slope (SS) and the 0-MHz Intercept (SI) parameters, as outlined in equations 3 and 4 [8]. The range for applying linear regression was previously determined when -6dB bandwidth values were acquired from the power spectrum of the reference scan.

S(f)=SSf+SI [3]

$$MBF=S(f_{c})$$ [4]

With *f_c_* being the transducer's centre frequency.

Additional ultrasound data analysis involved extraction of the backscatter coefficient (σ_*b*_) and estimation of scatterer properties based on the method established by Insana and Hall [55]. Briefly, the backscatter coefficient may be estimated from the normalized power spectrum using the equation:

$$\sigma_{b}\left(f\right)=\frac{1.45R^{2}}{A_{o}}S(f)$$ [5]

where *A_o_* is the area of the transducer aperture calculated from *A_o_* = *πr*^2^ (r being the radius of the aperture), and R is the on-axis distances between the transducer and the proximal surface of the region of interest. The backscatter coefficient may then be related to scatterer properties through the equation:

$$\sigma_{b}\left(k\right)=\frac{1}{9}\;k^{4}\;a^{6}\;\bar{n}\gamma_{o\;}^{2}\;F(k,a)$$ [7]

where k is the wavenumber equal to $\frac{2\pi f}{c}$, with c being the speed of sound. The estimated acoustic concentration is described by the term $\bar{n}\gamma_{o}^{2}$, which is a product between the volumetric number density ($\bar{n}$) and the square of the relative impedance mismatch between the scatterer and surround media ($\gamma_{o}^{2}$). F(*f, a*) is the form factor which describes the change in shape of the backscatter coefficent as a function of frequency (*f*) and the scatter diameter (*a*). For the Anderson Fluid-filled sphere model [56] used in this study, the form factor, as derived from Insana et al. [57], is expressed by:

$$F\left(2k\right)={\left[\frac{J_{1}\left(2ka\right)}{\frac{2}{3}ka}\right]}^{2}$$ [8]

with k being the wavenumber, *a* being the estimated scatterer diameter, and *J_1_* describing a spherical Bessel function of the first kind and first order. Given this form factor and the estimated backscatter coefficient, the estimated scatterer diameter may be acquired through minimization of the average standard deviations between estimated and theoretical backscatter coefficients, expanded on in Insana at el. [57]. Once *a* is acquired, the estimated acoustic concentration may be determined through Eq. 7

### Transmission electron microscopy

Centrifuged cell samples were fixed in 2.5% (w/v) glutaraldehyde (Fischer Scientific, Mississauga, ON) with 0.1M sodium cacodylate buffer (Electron Microscopy Sciences, Hatfield, PA) for 48 hours at 4°C, and then stained with 1% osmium tetroxide. Samples were dehydrated then polymerized and imaged. Imaging was carried out using a Zeiss EM902 electron microscope operating at 80 kV energy and at 10000x magnification for imaging whole cells and 100000x magnification for imaging chromatin clusters and 30 nm strands.

## SUPPLEMENTARY MATERIALS FIGURES



## Abbreviations

AML: Acute Myeloid Leukemia
CT: Computed Tomography
DNA: Deoxyribonucleic Acid
HFUS: High-frequency Ultrasound
MBF: Mid-band fit
MRI: Magnetic Resonance Imaging
PET: Positron Emission Tomography
RECIST: Response Evaluation Criteria in Solid Tumors
RF: Radiofrequency
ROI: Region of Interest
TEM: Transmission Electron Microscopy.
