# Supplementary material for: Effect of chromatin structure on quantitative ultrasound parameters
Source: Oncotarget. 2017 Jan 25;8(12):19631–44. doi: 10.18632/oncotarget.14816 (PMC5386710; doi:10.18632/oncotarget.14816)
Supplement: Supplementary file 1 [file oncotarget-08-19631-s001.pdf]

Effect of chromatin structure on quantitative ultrasound parameters

SUPPLEMENTARY FIGURES

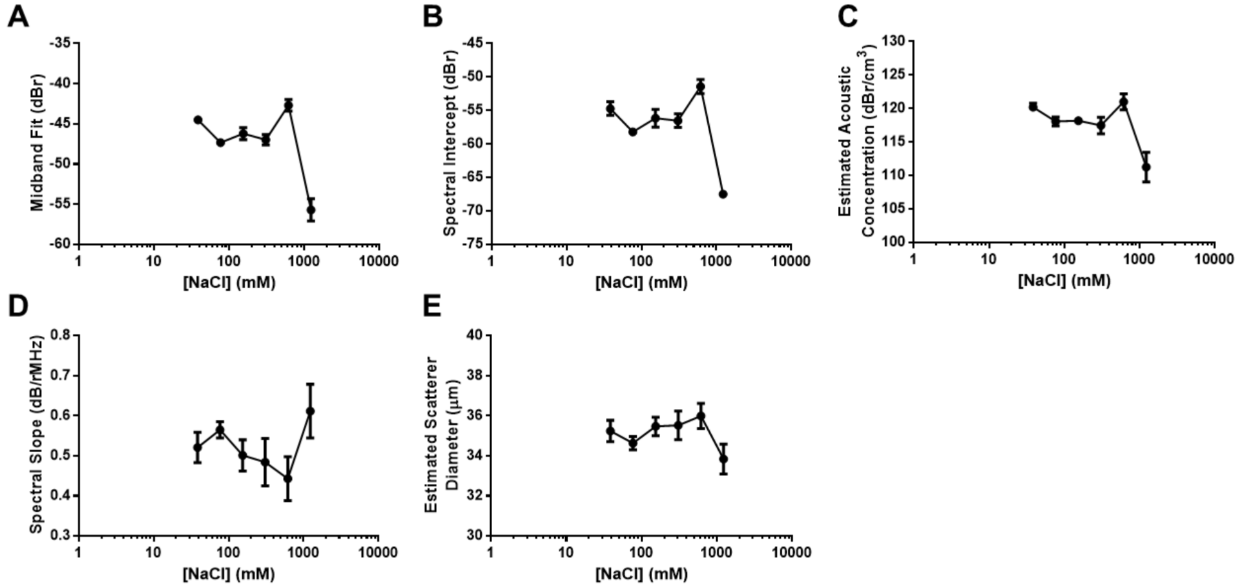

Supplementary Figure 1: Spectral parameter and form factor results from isolated nuclei subjected to salinities ranging from 1/4X to 8X physiological sodium concentration. A. Midband fit, **B.** spectral intercept, and **C.** estimated acoustic concentration trends in isolated nuclei were similar to those observed for whole cells. The notable exception was that decreases in these parameters are shown to occur at the lower 8X concentration. **D.** Spectral slope and **E.** estimated acoustic diameter did not change significantly until 8X NaCl concentration, for which decrease in scatterer size correspond to cellular and organelle shrinkage under hypertonic conditions. Error bars represent SD at n ≥ 3 for all conditions.

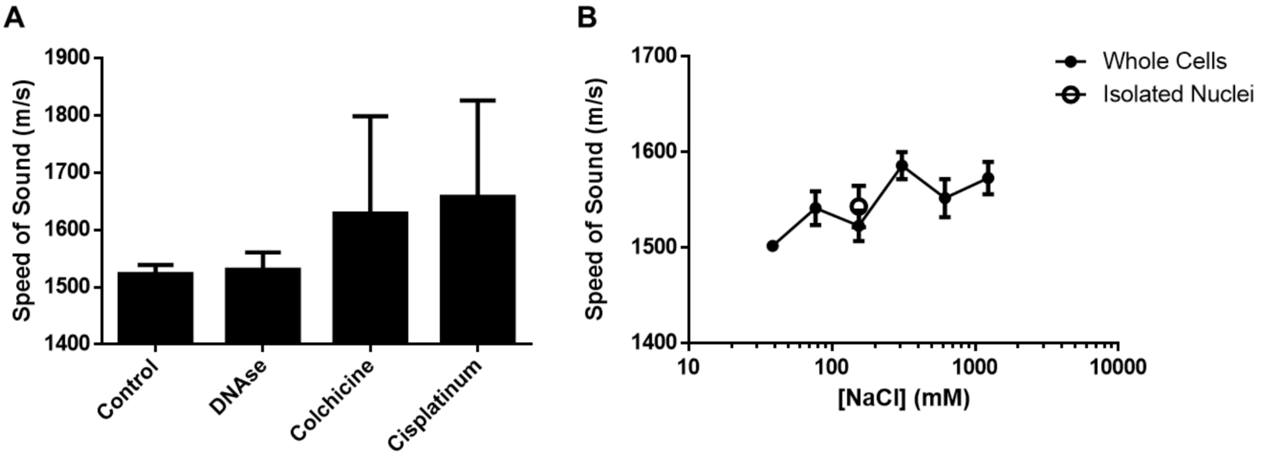

Supplementary Figure 2: Speed of sound data for treatments investigated for A. DNase I, Colchicine, and Cisplatinum treatments and B. alterations of sodium concentration for whole cells and isolated nuclei. For all conditions and for comparison of whole cells to isolated nuclei, no statistically significant changes were observed, indicating that corresponding changes to spectral parameters did not result from bulk changes to the speed of sound. Error bars represent SD at n ≥ 3 for all conditions.
